# Coexistence of **KRAS** and *BRAF* Mutations in Colorectal Cancer: A Case Report Supporting The Concept of Tumoral Heterogeneity

**DOI:** 10.22074/cellj.2017.5123

**Published:** 2017-05-17

**Authors:** Pegah Larki, Ehsan Gharib, Mohammad Yaghoob Taleghani, Fatemeh Khorshidi, Ehsan Nazemalhosseini-Mojarad, Hamid Asadzadeh Aghdaei

**Affiliations:** 1 Basic and Molecular Epidemiology of Gastrointestinal Disorders Research Center, Research Institute for Gastroenterology and Liver Diseases, Shahid Beheshti University of Medical Sciences, Tehran, Iran; 2 Gastroenterology and Liver Disease Research Center, Research Institute for Gastroenterology and Liver Diseases, Shahid Beheshti University of Medical Sciences, Tehran, Iran

**Keywords:** *BRAF*, CRC, EGFR, *KRAS*

## Abstract

The detection of *KRAS* and *BRAF* mutations is a crucial step for the correct therapeutic approach and predicting the epidermal growth factor receptor (EGFR)-targeted therapy resistance of colorectal carcinomas. The concomitant *KRAS* and *BRAF* mutations occur rarely in the colorectal cancers (CRCs) with the prevalence of less than 0.001% of the cases. In patients with *KRAS*-mutant tumors, *BRAF* mutations should not regularly be tested unless the patient is participating in a clinical trial enriching for the presence of *KRAS* or *BRAF*-mutated tumor. The current report demonstrates a case with advanced adenocarcinoma of the colon showing the coexistence of *KRAS* and *BRAF* mutations and may have profound clinical implications for disease progression and therapeutic responses.

## Introduction

Colorectal cancer (CRC) is one of the most common cancers, especially in the developed countries and its worldwide mortality rates exceed 700,000 deaths per year. CRC usually causes by the mutations in the epithelial cells of the gastrointestinal surfaces resulting in hyperactivity of the signaling pathways and finally transforms these cells into the adenomatous polyps. Accumulation of the inherited or acquired mutations transits the adenomatous polyps to malignancy. The genetic mutations and morphological changes of this progression path, have been well described in the previous studies (1,2). Significant genetic heterogeneity implicated in the CRC tumors, varying from the heterogeneous somatic DNA mutations to the chromosomal imbalances and even epigenetic factors such as DNA methylation (3). The current consensus theory of the heterogeneity of the CRC tumors has been underscored in most researches and highlighting the differences of the mutational status of *KRAS* gene between the primary and metastatic tumors (3-5). 

*KRAS* protein is a key factor in the regulation of cell mitosis. *KRAS* activates its primary downstream target protein named *BRAF*, a serine- threonine protein kinase that mediates the *KRAS* signal toward the downstream effectors, Mitogen- activated protein (MAP) kinase extracellular signal-regulated kinase (MEK) and the extracellular signal-regulated kinase (ERK) (6). In normal situations, wild-type *KRAS* provokes cell cycle progression. However, it could act as a gate keeper throughout tumor growth and block cancer development (7). Mutations in the gene encoding *KRAS*, would change its ability in hydrolyzing GTP to GDP and results in constantly activated *KRAS* protein, as previously reported (8,9). 

The impact of *KRAS* abnormal activity in cancer cells invasion has been well described by Liao et al. (10) as permanent activated *KRAS*, upregulates the metalloproteinase 2 (MMP2) expression level, and may promote cancer aggressiveness. Also, the elevated level of mutated *KRAS* enhances the carcinoembryonic antigen (CEA) level and blocks the monolayer formation of colon epithelial cells (11). The mutations in *KRAS* and *BRAF* genes as the key mediators in the epidermal growth factor receptor (EGFR) signaling pathways play an important role in the colorectal pathogenesis and are associated with the primary resistance to the EGFR inhibitors (12-15). *KRAS* mutations as one of the most and best- described prognostic factors in the prediction of the resistance to EGFR-targeting therapeutic agents, have been reported in nearby 40% of the patients affected with the CRC (16-20). The vast majority of the *KRAS* mutations occur in the codons 12 and 13 of the exon 2, whereas the remainders occur mostly in the codons 61 and 146. *BRAF* mutations have been recently reported in 5 to 15% of CRC cases (21-23). Beside the *KRAS* mutations, it has recently been shown that there is a similar association between the *BRAF* mutations and resistance to EGFR-targeting agents in CRC patients (17,24,25). However, the likelihood of the presence of two or more mutations in the same codon of the *KRAS* gene is too rare and the reports of these co-occurrence mutations have little been documented in colorectal cancer. In addition, the clinical consequences and prognostic values of the multiple mutations in the CRC patients have not been fully elucidated yet (15,26-28). Up to now, the joint mutant alterations in *KRAS* and *BRAF* sequences in a patient seem very rare (17). However, this study reports a case of the coexistence of the two somatic mutations in the codon 12 of the exon 2 of the *KRAS* gene and the codon V600 of the *BRAF* gene in a 50-year-old man affected with an advanced adenocarcinoma of the rectum. This supports the possibility of the colorectal tumors originated from two clonal origins with the coexistence of different mutations at the same genetic level. 

## Case report

A 50-year-old male patient suffering from the tenesmus and the presence of the blood in his stool, was admitted to the Gastroenterology and Liver Disease Research Institute, Taleghani Hospital, Tehran, Iran in January 2013. He referred with no personal or family history of the colorectal carcinoma or other related gastrointestinal diseases. Following the colonoscopy procedure, a hyperemic and flat lesion in the descending colon was discovered. The colon biopsy was performed to evaluate the depth of invasion in colon carcinoma in combination with the clinical examination by computed tomography scan. The final diagnosis was advanced colon cancer with liver metastases T3N2M1, stage IV. 

After obtaining informed consent from patient, the EGFR immunohistochemical expression profile was investigated by anti-EGFR monoclonal antibody according to the manufacturer’s descriptions. The paraffin-embedded tissue sections were collected on microscopic slides. Tumor and tumor-free areas of the samples were identified within 15 μm thick deparaffinized, lightly hematoxylin-counterstained sections and subjected to the pathological studies. Each microdissected area was applied to the DNA extraction followed by the mutational analysis of *KRAS* (exons 2 and 3) and *BRAF* (exon 15) by using polymerase chain reaction (PCR) and direct sequencing. PCR cycling conditions were performed according to the manufacturer’s instruction. The ABI 3130 genetic analyzer (Applied Biosystems, USA) and the Big Dye Terminator (Applied Biosystems, USA) were used for the sequencing reactions of this study. 

To investigate microsatellite instability (MSI), the DNA samples from the blood and microdissected tumoral areas of the patient were examined by using the BAT26, BAT25, NR24, NR21 and NR27 mononucleotide microsatellite markers. These markers are known to be the most sensitive markers of MSI status and widely uses for identification of the CRC cases with the concomitant mismatch repair (MMR) defects. 

*KRAS* and *BRAF* mutational status analysis was performed on the representative lesion of the colon, using the PCR-sequencing method and MSI analysis. The identified mutations in *KRAS* and *BRAF* genes were a mutation in codon 12 of exon 2 and a missense nucleotide base change in codon 600 of exon 15 (GTG to GAG), respectively. However, MSI was not detected. 

## Discussion

CRC is estimated as the third commonly diagnosed type of cancer and the fourth common cause of the cancer mortality after the lung, stomach and liver cancers, in both males and females. Considering the invention of new therapeutics including Irinotecan and Oxaliplatin in addition to the development of targeted therapies such as Cetuximab and Bevacizumab, the survival rates of diagnosed patients have been significantly improved. It has been shown in several experiments that the *KRAS* mutations in primary colorectal tumors play a prognostic role as a predictor of resistance to the anti-EGFR antibodies (12,18,19). Although the results of the *KRAS* mutational analysis of the primary tumor usually match with the metastases in only 5 to 10% of the cases, the *KRAS* mutational status is heterogeneous between the primary tumors and the metastases (5,29-32). This observation may reflect the increased genetic instability in cells that progressively acquiring mutations or the presence of a heterogeneous group of the neoplastic cells inside the tumor (33,34). Moreover, the co-occurrence of *KRAS* and *BRAF* mutations in the same colorectal tumor has been reported in few studies. These studies have also reported the correlation of the concomitant *KRAS* and *BRAF* mutations with the clinical and morphological characteristics (15,26-28). 

Because of the rarity of this observation, it is not clear whether the concomitant *KRAS* and *BRAF* mutant tumors have a different biology and natural history than singly *KRAS* or *BRAF* mutant tumors, or which of the two mutations play the dominant role in driving the tumor proliferation. It has been noticed that the proportion of the concomitant mutations is associated with the degree of transmural invasion of the tumor; moreover, 2.8 and 9.4% of concomitant mutations occur in the T2 and T4 tumors, respectively, suggesting the activation role of both genes in the tumor progression (35). Molecular profiling studies proved that the *KRAS* and *BRAF* mutant tumors have different mutation results and probably depict the different over-activated signaling cascades (36). Currently, it is not obvious that which gene expression profile pattern is dominant (35). Considering the mutational characteristics of the CRC, including the high degree of tumor heterogeneity and the vast variety of the mutations along with the epigenetic alterations, it is likely that the gene expression profiling of these tumors will provide therapeutics benefits in terms of better understanding and determining activation of the different signaling pathways in tumors and applying the obtained prognostic biomarkers in the management and treatment of the disease. The current study supports the polyclonal and intratumoral heterogeneity features of the colon cancer, in which a mixture of cell clones with varying mutations characterizes the tumors. The coexistence of distinct clones with the high degree of the mutational heterogeneity could bring about profound clinical indications in terms of the disease progression, prognostic features and responses to particular therapeutic regimens. In particular, the present case appears to support the hypothesis that the co-occurrence of *KRAS* and *BRAF* mutations is associated with more aggressive clinical manifestations. 

## Conclusion

Currently, *KRAS* mutation status and microsatellite instability analysis are the only well-explained prognostic biomarkers as the negative predictors of the therapy efficiency in colorectal cancer. On the other hand, concomitant *KRAS* and *BRAF*-mutated colorectal tumors are relatively rare, so that the routine analysis of *BRAF* mutations in tumors with the *KRAS* mutations is not recommended. The case shows, that the concomitant *KRAS* and *BRAF* mutations is associated with more severe disease. This may provide clinical implications for cancer progression and management. Our case study suggests that the analysis of *BRAF* mutation, especially in *KRAS*-positive tumors would be highly advisable. In addition, the future clinical trials related to colorectal cancer, should specifically consider the eligibility of patients with concomitant *KRAS* and *BRAF*-mutated tumors. 
